# Application of latent class analysis in diagnosis of graft-versus-host disease by serum markers after allogeneic haematopoietic stem cell transplantation

**DOI:** 10.1038/s41598-020-60524-2

**Published:** 2020-02-27

**Authors:** Maedeh Amini, Anoshirvan Kazemnejad, Aliakbar Rasekhi, Farid Zayeri, Abbas Hajifathali, Farzaneh Tavakoli

**Affiliations:** 1 Department of Biostatistics, Faculty of Medical Sciences, Tarbiat Modares University, Tehran, Iran; 2 Proteomics Research Center and Department of Biostatistics, Faculty of Paramedical Sciences, Shahid Beheshti University of Medical Sciences, Tehran, Iran; 3 Hematopoietic Stem Cell Research Center, Shahid Beheshti University of Medical Sciences, Tehran, Iran; 4 Taleghani Hospital, Shahid Beheshti University of Medical Sciences, Tehran, Iran

**Keywords:** Diagnostic markers, Statistics, Haematopoietic stem cells

## Abstract

Graft-versus-host disease (GVHD) is one of the major causes of morbidity and mortality in 25–70% of patients. The gold standard (GS) test to confirm the diagnosis of GVHD has some limitations. The current study was conducted to evaluate the accuracy of three serum markers in diagnosing GVHD without a GS. 94 patients who were hospitalized for allogeneic transplantation were studied. Mean levels from day of haematopoietic stem cell transplantation (HSCT) to discharge of serum uric acid (UA), lactate dehydrogenase (LDH), and creatinine (Cr) were measured for all participants. We adapted a Bayesian latent class analysis to modelling the results of each marker and combination of markers. The Sensitivity, Specificity, and area under receiver operating characteristic curve (AUC) for LDH were as 51%, 81%, and 0.70, respectively. For UA, the Sensitivity, Specificity, and AUC were 54%, 75%, and 0.71, respectively. The estimated Sensitivity, Specificity, and AUC of Cr were 72%, 94%, and 0.86, respectively. Adjusting for covariates, the combined Sensitivity, Specificity, and AUC of the optimal marker combination were 76%, 83%, and 0.94, respectively. To conclude, our findings suggested that Cr had the strongest diagnosis power for GVHD. Moreover, the classification accuracy of the three-marker combination outperforms the other combinations.

## Introduction

Hematopoietic stem cell transplantation (HSCT), either autologous or allogeneic, is an intensive therapy for patients who suffer from refractory or relapsing neoplastic disease and non-neoplastic genetic disorders^1,2^. Global, the rate of allogeneic HSCT continues to increase such that about 50,000–60,000 transplantation was performed annually. HSCT is a very complex medical procedure that associated with different types of comorbidities threatening the patient life^3^. During the last few decades, substantial progress has been made with regard to HSCT management due to the improved diagnostic procedures and novel therapies. In HSCT success is limited by two main factors: transplant-related morbidity or mortality (TRM) and disease relapse^4^. Graft-versus-host disease (GVHD) is the major cause of TRM which classified as either acute GVHD (aGVHD) or chronic GVHD (cGVHD)^2^. Clearly, GVHD is a common serious complication of HSCT in which immune cells from the donor attack recipient non-hematopoietic tissues, containing the gastrointestinal tract, liver, skin, and lungs^5^. Moreover, GVHD occurs in 30 to 50% of HLA-matched sibling transplants and up to 60 to 90% of mismatched transplants^6^. Accordingly, it is important to monitor the condition with timely diagnosis to avoid irreversible damage.

Biopsy as a gold standard (GS) test, is known for confirming the diagnosis of GVHD and should be used when there are competing differential diagnoses^7^. Nevertheless, challenges for biopsy includes the risk of hematoma and bleeding, difficulty to perform in some ulcerated areas, and its invasive nature^8,9^. Nowadays, the diagnosis of GVHD is usually based on clinical symptoms and signs while avoiding invasive tissue biopsies. Nonetheless, signs and symptoms of GVHD vary among individuals over time which makes the diagnosis of GVHD extremely challenging^10^. In recent years, candidate biomarkers were investigated through hypothesis-driven approaches that have important role in clinical diagnosis and may aid with prediction, prognostication, and monitoring therapeutic responses of GVHD^11^. Of note, if a biomarker was specific for diagnosis, delays in obtaining biopsies interpretation could be avoided. Lactate dehydrogenase (LDH), uric acid (UA), and creatinine (Cr) have been evaluated as chemical factors in prior studies in the field of transplantation, although no specific agreement has been reached on their roles^12,13^. The present article found that the analysis of these serum markers is simple and inexpensive method so, they might be employed to accelerate GVHD diagnosis. Further, these markers might have a postulated role in the pathophysiology of GVHD^14^.

To evaluate the performance of a diagnostic test or a biomarker, getting valid estimates of its accuracy is necessary. The accuracy means test’s ability to discriminate between patients who have the target condition and those who do not^15^. To analyze the accuracy of a single or set of diagnostic tests, the results of the tests in relation to the outcome of the GS (or reference standard) is examined. However, for many diseases a GS test does not exist or not applicable. In such circumstances, misclassification of the disease status and accordingly biased estimates of test performance and disease prevalence might be occur. To overcome the absence of a GS test, latent class models (LCMs) as a member of structural equation modeling (SEM) have been proposed to evaluate the accuracy of diagnostic tests. This modeling approach, involves treating the GS as an unobserved latent class and obtaining a model-based estimate of diagnostic accuracy, often with the intention of hierarchical modeling^16^. Of note, LCM can be utilized to combine the results of different tests into a diagnostic composite test to compare the accuracy of those tests when there is a lack of a GS^17^. Various authors claimed that LCMs can provide valid estimates of accuracy even without a GS test.

In our literature review, we found a few studies about the relationships between LDH, Cr, and UA levels and GVHD. Although valuable, these studies provide no information about assessing the diagnostic accuracy of the serum markers especially when a GS reference test information is not available. Thus, the outline of the rest of the present paper is twofold: (1) the Bayesian estimations of classification accuracy measures including Sensitivity, Specificity, and area under receiver operating characteristic curve (AUC) were obtained for each the diagnostic test; (2) We consider different combinations of the continuous tests outcomes to identify the optimal composite test for diagnosing of GVHD using Bayesian latent class models.

## Results

### Patient characteristics

A total of 94 subjects, 49 (52.1%) females and 45 (47.9%) males were examined. The mean (SD) age of participants was 35.06 ±10.31 years old (range 14–57). Also, the individuals had a mean (SD) body mass index (BMI), a mean (SD) LDH, a mean (SD) UA, and a mean (SD) Cr of 25.28 ± 4.41 kg/m2, 2.64 ± 0.14 U/L, 3.81 ± 0.87 mg/dL, and 0.90 ± 0.15 mg/dL, respectively. Likewise, the mean (SD) donor age of males and females was 34.23 ± 11.66 and 31.91 ± 9.23 years old, respectively.

### Evaluating diagnostic accuracy for each serum marker

**Figure 1:**
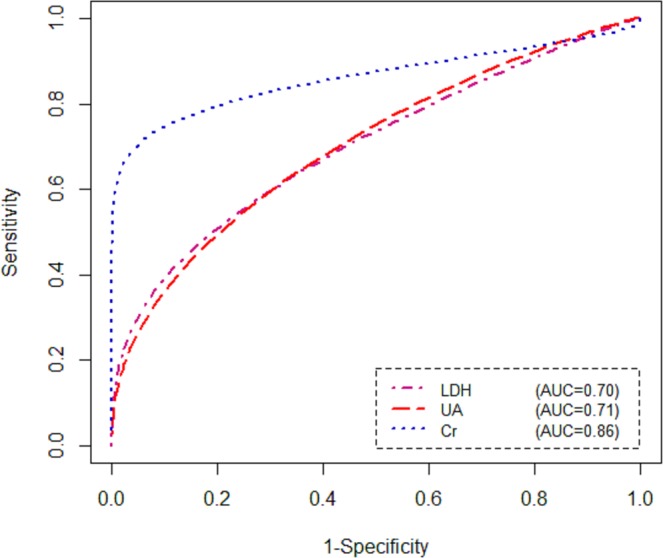
ROC curves for each serum marker without a gold standard test.

Table 1 presents the estimates of diagnostic accuracy parameters for each marker based on optimal cut-off points. As seen in Table 1, the mean of Sensitivity, Specificity, and AUC were 51% (95% CrI [0.45–0.58]), 81% (95% CrI [0.77–0.89]), and 0.70 (95% CrI [0.65–0.78]), respectively for LDH. Also, the Sensitivity, Specificity, and AUC for UA were estimates as 54% (95% CrI [0.50–0.58]), 75% (95% CrI [0.70–0.83]), and 0.71 (95% CrI [0.68–0.77]), respectively. Additionally, the estimated Sensitivity, Specificity, and AUC of Cr were 72% (95% CrI [0.68–0.80]), 94% (95% CrI [0.87–0.99]), and 0.86 (95% CrI [0.81–0.90]), respectively. Further, the estimated Δs for LDH, UA, and Cr were 0.11, 0.16, and 0.23, respectively. Figure 1 shows the ROC curves for LDH, UA, and Cr to diagnosis of GVHD in the absence of a GS.

**Table 1:** Bayesian diagnostic accuracy parameter estimates for each biomarker.

Biomarker	cut-off	Accuracy parameters	Mean	SD	Median	95% CrI
LDH	81.0					
		Sensitivity	0.51	0.12	0.51	(0.45–0.58)
		Specificity	0.81	0.11	0.82	(0.77–0.89)
		AUC	0.70	0.11	0.71	(0.65–0.78)
UA	74.0					
		Sensitivity	0.54	0.10	0.54	(0.50–0.58)
		Specificity	0.75	0.12	0.76	(0.70–0.83)
		AUC	0.71	0.10	0.72	(0.68–0.77)
Cr	49.0					
		Sensitivity	0.72	0.11	0.73	(0.68–0.80)
		Specificity	0.94	0.11	0.93	(0.87–0.99)
		AUC	0.86	0.10	0.85	(0.81–0.90)

Notes: SD: standard deviation; CrI: credible interval; LDH: lactate dehydrogenase; UA: uric acid; Cr: creatinine; AUC: area under receiver operating characteristic curve.

### Evaluating diagnostic accuracy for combination of markers’ results

**Figure 2:**
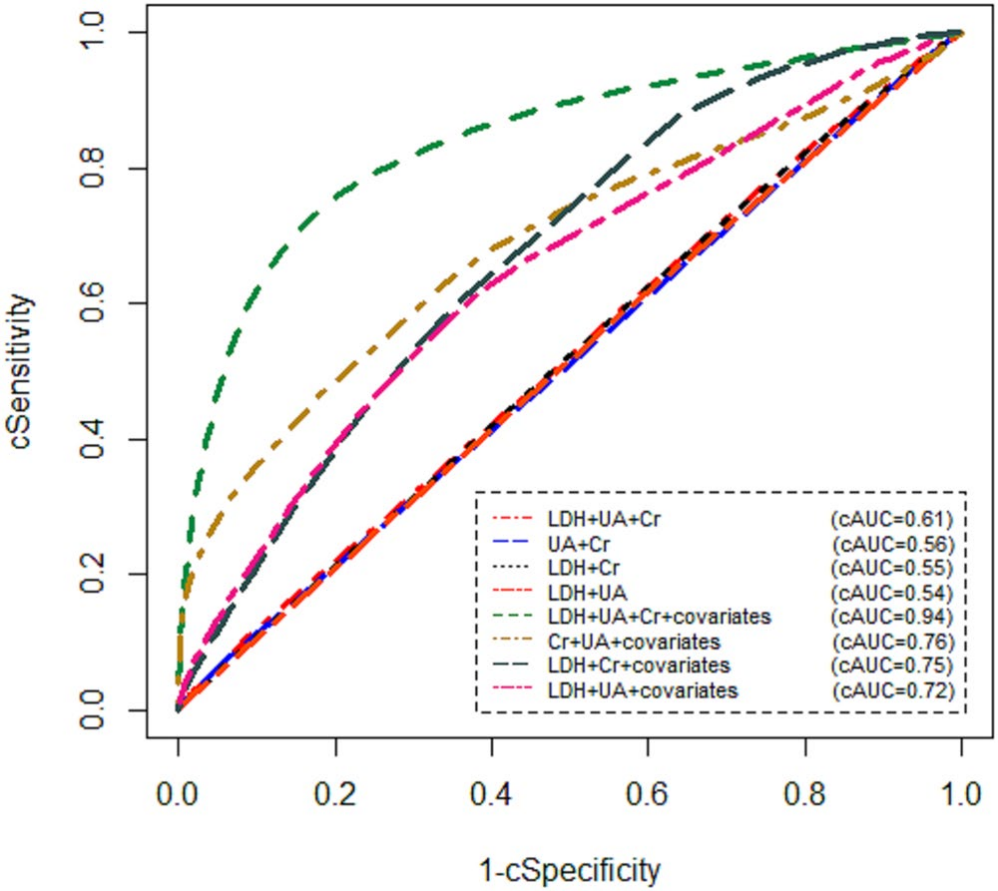
cROC curves for various combinations of the serum markers with and without covariate adjustment in the absence of a gold standard test.

Table 2 lists the Bayesian estimates of diagnostic accuracy parameters for various combination of markers based on the optimal cut-off points. According to Table 2, in combination of LDH and Cr results, the estimates of combined Sensitivity (cSen), combined Specificity (cSp), and cAUC were 57% (95% CrI [0.53–0.64]), 43% (95% CrI [0.39–0.52]), 0.55 (95% CrI [0.50–0.65]), respectively. Moreover, the cSen, cSp, and cAUC were estimated as 56% (95% CrI [0.51–0.60]), 41% (95% CrI [0.37–0.52]), and 0.54 (95% CrI [0.48–0.62]), respectively, for combination of LDH and UA values. In addition, the cSen, cSp, and cAUC for combination of UA and Cr, were estimated as 54% (95% CrI [0.50–0.65]), 46% (95% CrI [0.41–0.53]), and 0.56 (95% CrI [0.51–0.65]), respectively. Meanwhile, in combination of LDH, Cr, and UA results, the estimates of cSen, cSp, and cAUC were 58% (95% CrI [0.53–0.65]), 49% (95% CrI [0.43–0.52]), and 0.61 (95% CrI [0.57–0.68]), respectively. Table 3 reports the Bayesian estimates of diagnostic accuracy indices for each combination of the markers’ results adjusting for age, DP-gender, GVHD prophylaxis, and conditioning regimen according to the optimal cut-off points. Based on Table 3, the cSen, cSp, and cAUC were estimated as 61% (95% CrI [0.57–0.71]), 63% (95% CrI [0.59–0.72]), and 0.75 (95% CrI [0.68–0.79]), respectively, for combining LDH and Cr results. Likewise, the cSen, cSp, and cAUC for combination of LDH and UA values were 62% (95% CrI [0.58–0.71]), 67% (95% CrI [0.62–0.71]), and 0.72 (95% CrI [0.67–0.78]), respectively. Further, in combination of Cr and UA results, the cSen, cSp, and cAUC were estimated as 60% (95% CrI [0.54–0.68]), 65% (95% CrI [0.60–0.74]), and 0.76 (95% CrI [0.71–0.84]), respectively. Finally, the estimated cSen, cSp, and cAUC of combination of all the three markers were 76% (95% CrI [0.71–0.80]), 83% (95% CrI [0.78–0.89]), and 0.94 (95% CrI [0.90–0.99]), respectively. Figure 2 displays the ROC curves for different combinations of the markers with and without covariate adjustment. According to optimal marker combination (i.e., combining LDH, Cr and UA values adjusting for the covariates), of 94 participants, about 43 (45.7%) subjects were assigned to GVHD group. Moreover, about 51 (54.3%) subjects of 94 participants were GVHD-free. Table 4 summarises the demographic characteristics of the study population across GVHD groups. As seen in Table 4, the GVHD group had significantly higher levels of LDH and Cr than in those with no GVHD (2.68 ± 0.11 vs. 2.62 ± 0.14, p = 0.02; 0.95 ± 0.16 vs. 0.87 ± 0.13, p = 0.02, respectively). In contrast, we found no significant differences in means UA, age, and BMI between with and without GVHD groups (p = 0.40, p = 0.86, p = 0.92, respectively). In addition, no statistically significant relationship was found between categorical variables and GVHD (p > 0.05).

**Table 2:** Bayesian diagnostic accuracy parameter estimates for various combinations of the biomarkers without covariate adjustment.

Composite test	cut-off	Accuracy Parameters	Mean	SD	Median	95% CrI
LDH and Cr	31					
		cSensitivity	0.57	0.11	0.58	(0.53–0.64)
		cSpecificity	0.43	0.12	0.46	(0.39–0.52)
		cAUC	0.55	0.14	0.56	(0.50–0.65)
LDH and UA	11					
		cSensitivity	0.56	0.10	0.55	(0.51–0.60)
		cSpecificity	0.41	0.14	0.43	(0.37–0.52)
		cAUC	0.54	0.13	0.55	(0.48–0.62)
UA and Cr	10					
		cSensitivity	0.54	0.14	0.58	(0.50–0.65)
		cSpecificity	0.46	0.11	0.47	(0.41–0.53)
		cAUC	0.56	0.13	0.55	(0.51–0.65)
UA and Cr and LDH	32					
		cSensitivity	0.58	0.12	0.57	(0.53–0.65)
		cSpecificity	0.49	0.10	0.48	(0.43–0.52)
		cAUC	0.61	0.11	0.62	(0.57–0.68)

The calculation of cut-off points for the markers’ combinations is as the same as one marker. In fact, by combining the markers’ results, only one composite test is obtained. Notes: SD: standard deviation; CrI: credible interval; LDH: lactate dehydrogenase; UA: uric acid; Cr: creatinine; cSensitivity: combined Sensitivity; cSpecificity: combined Specificity; cAUC: combined area under receiver operating characteristic curve.

**Table 3:** Bayesian diagnostic accuracy parameter estimates for various combinations of the biomarkers with covariate adjustment.

Composite test	cut-off	Accuracy Parameters	Mean	SD	Median	95% CrI
LDH and Cr	36					
		cSensitivity	0.61	0.14	0.63	(0.57–0.71)
		cSpecificity	0.63	0.13	0.64	(0.59–0.72)
		cAUC	0.75	0.11	0.73	(0.68–0.79)
LDH and UA	30					
		cSensitivity	0.62	0.12	0.64	(0.58–0.71)
		cSpecificity	0.67	0.10	0.66	(0.62–0.71)
		cAUC	0.72	0.11	0.73	(0.67–0.78)
UA and Cr	27					
		cSensitivity	0.60	0.14	0.62	(0.54–0.68)
		cSpecificity	0.65	0.12	0.66	(0.60–0.74)
		cAUC	0.76	0.12	0.77	(0.71–0.84)
UA and Cr and LDH	47					
		cSensitivity	0.76	0.10	0.75	(0.71–0.80)
		cSpecificity	0.83	0.11	0.84	(0.78–0.89)
		cAUC	0.94	0.10	0.94	(0.90–0.99)

The calculation of cut-off points for the markers’ combinations is as the same as one marker. In fact, by combining the markers’ results, only one composite test is obtained. Notes: SD: standard deviation; CrI: credible interval; LDH: lactate dehydrogenase; UA: uric acid; Cr: creatinine; cSensitivity: combined Sensitivity; cSpecificity: combined Specificity; cAUC: combined area under receiver operating characteristic curve.

**Table 4:** Demographic and clinical characteristics of patients across the GVHD groups.

Characteristics	GVHD (n = 43)	Non- GVHD (n = 51)	P value
LDH (U/L), mean (SD)	2.68 (0.11)	2.62 (0.14)	0.028^*^
UA (mg/dl), mean (SD)	3.91 (0.87)	3.76 (0.87)	0.401^*^
Cr (mg/dL), mean (SD)	0.95 (0.16)	0.88 (0.13)	0.020^*^
Age (years), mean (SD)	35.20 (9.86)	34.82 (11.19)	0.866^*^
Donor age (years), mean (SD)	35.32 (12.53)	31.77 (9.04)	0.177^*^
BMI (kg/m^2^), mean (SD)	25.22 (4.0)	25.31 (4.65)	0.927^*^
**Gender, n (%)**			0.459^**^
Male	18 (52.9)	27 (45.0)	
Female	16 (47.1)	33 (55.0)	
**Smoking, n (%)**			0.377^**^
Never	29 (85.3)	52 (88.1)	
Past	5 (14.7)	5 (8.5)	
Current	0 (0)	2 (3.4)	
**GVHD Prophylaxis, n (%)**			0.214^**^
CSA+MTX	27 (79.4)	54 (90.0)	
CSA+MTX+ATG	7 (20.6)	6 (10.0)	
**Conditioning regimen, n (%)**			0.515^**^
Bu-Cy	19 (55.9)	34 (56.7)	
Bu-Fu	9 (26.5)	20 (33.3)	
Bu-Fu-ATG	6 (17.6)	6 (10.0)	
**Blood Group, n (%)**			0.280^**^
A	11 (32.4)	15 (25.9)	
B	11 (32.4)	11 (19.0)	
AB	5 (8.7)	10 (17.2)	
O	9 (26.5)	22 (37.9)	
**DP-Gender, n (%)**			0.256^**^
M-M	5 (14.7)	14 (23.3)	
M-F	13 (38.2)	19 (31.7)	
F-F	3 (8.8)	12 (20.0)	
F-M	13 (38.2)	15 (25.0)	

The groups of GVHD (with and without) were based on the optimal composite test (i.e., combination of Cr, LDH, and UA adjusting for age, DP-gender, GVHD prophylaxis, and conditioning regimen). GVHD: graft-versus-host disease; SD: standard deviation; LDH: lactate dehydrogenase; UA: uric acid; Cr: creatinine; BMI: body mass index; CSA: cyclosporine A; MTX: Methotrexate; ATG: anti-thymocyte globulin; Bu: busulfan; Cy: cyclophosphamide; Fu: fludarabine; M: male; F: female; * T-test; ^**^χ^2^.

## Discussion

According to the results of latent class models for each marker, Cr performed better than the other markers for classification, which achieved an AUC of 0.86 (Sensitivity = 72%, Specificity = 94%). More clearly, 72% of patients with GVHD could be correctly diagnosed by measuring serum Cr. Ruutu *et al*. reported that one of the laboratory signs of post-transplant-microangiopathy is high creatinine^18^. Some previous studies have provided evidence on the association between GVHD and angiopathy. Evaluation of two markers of angiopathy from the angiopoietin family has shown that their levels in patients with GVHD are different from those without GVHD^19^. Since evaluation of angiopathic markers, including vascular endothelial growth factor, requires specific kits, creatinine may be used as an alternative factor.

Sometimes the clinicians are faced with the question of combining information from multiple diagnostic tests in an efficient manner. We investigated whether combining the values of UA, LDH, and Cr likely will provide a more accurate diagnosis of GVHD and discrimination of cases and controls. In the case of combination of the serum markers without covariate adjustment, none of the double combinations have a good classification performance. In fact, the estimated cSensitivity, cSpecificity, and cAUC values were low and less than 0.60. Nevertheless, although cAUC of combining the three serum markers was sufficient, only 58% of patients with GVHD could be correctly detected. It means that addition of a third marker conferred no added benefit. Hence, we examined the effect of selected covariates including age, DP-gender, GVHD prophylaxis, and conditioning regimen on diagnostic power of linear marker combinations. Our findings indicated that the use of LDH, UA, and Cr combination was superior to 2-marker combinations in detection of GVHD. Indeed, this combination discriminates best between patient groups (GVHD and no-GVHD) due to the highest AUC value. On the other hand, the combining of the three markers’ results had the highest cSensitivity value such that 76% of patients with GVHD can be correctly detected. Furthermore, this combination with the highest cSpecificity can be correctly detected 83% of subjects without GVHD. It is thus important to point out that the combination of the three markers as an optimal composite test can diagnose GVHD more accurately. In essence, this combination may provide important insights for GVHD diagnosis and treatment efficacy assessment after HSCT. UA is released after conditioning and radiochemotherapy and can exacerbate inflammation which is considered one of the initiators of GVHD^20,21^. On the other hand, because UA is a danger signal, it can induce inflammation by activating the innate and adaptive immunity^22^. Further, serum LDH level could be a general indicator of tissue damage. Though a few studies have reported on the association between serum levels of LDH and UA values with GVHD, they failed to report the diagnostic accuracy parameters estimates individually. For example, Song *et al*. studied the influence of LDH level on the incidence of aGVHD after allogeneic stem cell transplantation. They found that the LDH levels was associated with the incidence of aGVHD using cox regression analysis^23^. Furthermore, Yeh *et al*. identified that the serum level of uric acid is associated with the development of aGVHD^24^. Likewise, Ostendorf *et al*. have indicated that UA was a significant predictor of aGVHD by multivariate analysis^12^. In addition, Joo *et al*. suggested that HSCT may significantly associated with changes in the serum UA levels in patients with hematologic disorders^25^. Notably, no study has identified the diagnostic efficacy of combination of the serum markers for GVHD diagnose.

In the current article, in order to overcome the problem of estimating diagnostic accuracy parameters when the true disease status is unknown, we applied a Bayesian latent class model. Besides, because there is no substantial overlap between the diseased and non-diseased groups for each chemical factor regarding Δs, we can conclude that the model works well.

In this study, we investigated whether the addition of the selected covariates to marker combinations and disease status could impact the magnitude or diagnostic accuracy. Based on our results, we concluded that this can improve discriminatory ability between two groups of individuals, cases and controls. Previously, some literature addressed the effect of covariate adjustment on classification performance of marker combinations. Similar findings were noted by them, where they found that combining the marker and covariate information can improve sensitivity, specificity, and AUC as well as improve the diagnostic performance^26,27^. More importantly, several factors have been introduced as GVHD risk factors of which the patients age is considered to have an influencing role in GVHD^28^. Further, according to the literature review, a number of studies have been demonstrated that gender disparity influence the GVHD incidence. For instance, Gahrton suggested that males receiving transplant from a female donor, are at greater risk of developing chronic GVHD and transplant related mortality than others^29^. Also, it is worth to note that the intensity of the conditioning regimen is highly effective in the incidence of GVHD^30^. Effect of conditioning regimen component on GVHD have been evaluated in numerous studies and it has been shown that adding some agents such as anti-thymocyte-globulin could reduce the risk of GVHD efficiently^31^.

Generally, recent studies have been focused on choosing a panel as GVHD markers and some have shown promising results^32–34^. However, some of these panels contain serum markers that require expensive commercial kits that are not routinely available in many countries. Therefore, we tried to suggest a panel that could be routinely measured. Since the incidence of GVHD depends on several factors, considering other conditions such as age and chemotherapy regimen can lead to more realistic results compared with evaluating only one marker regardless the underlying factors.

On the whole, the main methodological strength of this study is the use of latent class modelling technique. This helps to provide estimates of accuracy parameters for diagnostic tests incorporated in the model. Moreover, LCM is a flexible method which can incorporate different types of test outcomes. Importantly, using the Bayesian method for estimating the model parameters considering the small sample size was another advantage of the current paper. Nonetheless, further larger-scale studies are required in order to validate the candidate serum marker and optimal combination for routine application. It must be acknowledged that some limitations needed to address in the study. First, the primary limitation of this study was its cross-sectional design. This matter may cause that the causal inferences cannot be made. Second, the generalization of the results may be affected by the relatively small sample size. Third, the estimation of out-of-sample performance for the diagnostic accuracy parameters was not possible in this study. Forth, although the serum markers investigated in our study have the advantage of easy measurement, they may not have the ideal specificity because different factors may change their levels. For instance, LDH level may alter in hemolysis or viral situation. In addition, UA level could be influenced by diet, kidney function and some medications. Finally, Cr level depends on glomerular filtration rate and body mass. The final limitation is that the optimal cut-off points for each marker and combination of them were based on the model parameters’ estimates and had no clinical meaning. Thus we recommend that the clinicians investigate this topic in future research.

To the best of our knowledge, this is the first study to apply the Bayesian LCM approach to evaluate the accuracy of LDH, Cr, and UA for detecting GVHD without a perfect reference standard test. Indeed, the current paper attempted to classify GVHD based on the serum markers’ values. We concluded that Cr had the strongest diagnosis power for identifying GVHD in recipients of HSCT compared with use of another individual tests. Likewise, adjusting for age, DP-gender, GVHD prophylaxis, and conditioning regimen, our results suggest that the combination of serum LDH, UA, and Cr values provided a certain extent accuracy for discrimination of GVHD which performance was better than that of any other single marker or combination markers in the absence of a GS reference test. In short, either Cr individually or Cr combined with LDH and UA may serve as the indicator of diagnosing GVHD with high level of accuracy after HSCT.

## Materials and Methods

### Data source and study population

In present research, we used the allogeneic stem cell transplantation data which have been collected between August 2009 and March 2018. The medical information of 108 patients who underwent their first allogeneic stem cell transplantation during the post-transplant period was recorded at hematopoietic stem cell research centre related to Taleghani hospital affiliated to Shahid Beheshti University of Medical Sciences in Tehran, Iran. Covariates data including age, donor age, body mass index (BMI), blood group, the status of donor/patient (DP)-gender, conditioning regimen, and GVHD prophylaxis were extracted from medical records. Written informed consent was obtained from all patients. In addition, since one of the patients’ age was under 18 years, informed consent was provided from parents for study participation. This study was approved by Shahid Beheshti University of Medical Sciences ethics committee. All methods of the current study were performed in accordance with the relevant guidelines and regulations.

### Conditioning regimens

Myeloablative conditioning regimen (MAC) consisted of busulfan (0.8 mg/kg every 6 hours for 4 days, intravenously) followed by either cyclophosphamide (60 mg/kg/day for 2 days) or fludarabine (30 mg/m^2^ of body surface area once a day for 5 days). Regimen divided into three groups, including busulfan and cyclophosphamide, busulfan and fludarabine, anti-thymocyte globulin (ATG). For Hodgkin’s disease (HD) and non-Hodgkin’s lymphoma (NHL), reduced intensity conditioning (RIC) regimen comprised of fludarabine (30 mg/m^2^ of body surface area for 5 days, IV), Lomustine or CCNU (100 mg/m^2^ for 2 days, P.O) and melphalan (40 mg/m^2^ for 1 day, IV) was used^35^.

### GVHD prophylaxis

All patients received cyclosporine A (CSA) and methotrexate (MTX) for GVHD prophylaxis. A daily dose of 3 mg/kg/day CSA was administrated IV from day −2 until +5 and 12.5 mg/kg/day P.O. until day +180. Methotrexate (MTX) was administrated from day +1 with dose of 10 mg/kg IV and in days +3, +6 and +11 with the dose of 6 mg/kg, IV in combination with CSA. Some patients also received 2.5 mg/kg of ATG for 2 days (−1 and −2).

### Transplantation procedure

The stem cell source for all patients was mobilized peripheral blood. Granulocyte-colony stimulating factor (G-CSF) was administered subcutaneously at dosage of 5–10 μg/kg/day, for 4–5 consecutive days. The enumeration of donor peripheral blood CD34+ cell was performed by flow cytometry (Attune NxT, Invitrogen Life Technologies, Carlsbad, CA) on day 5 post G-CSF administration using PE-conjugated human anti-CD34 (PE-conjugated, EXBIO, Czech Republic) to determine the optimal day for apheresis. Plasma reduction for ABO minor-mismatched and red blood cell (RBC) depletion for major and bidirectional mismatched grafts were performed on the apheresis product. RBC depletion was performed using hydroxyl ethyl starch (HES) 6% (GRIFOLS, Spain). The number of CD34+ cells and CD3+ (FITC-conjugated, Beckman Coulter, Miami, FL) cells in apheresis product were counted and viability tests on all apheresis yields were performed using Trypan Blue viability dye (Biowest, France) before transplantation^36,37^.

### Laboratory tests

Peripheral blood samples were collected and UA levels in serum samples were evaluated using an ADVIA 1800 clinical chemistry analyser (Toshiba, Tochigi, Japan). Serum LDH and Cr level was measured on a Hitachi911 automatic analyser (Roche Diagnostics, Meylan, France). For all the three serum markers, mean levels from day 0 to discharge day was calculated. In fact, elevated the markers can be detected at mean levels during the time period after HSCT^38^.

### Statistical analysis

Before data analysis, we observed several extreme observations (outliers) for some variables (e.g., LDH) which can affect the results and create great difficulty in analysis. Hence, to prevent distortion of results, they should be discarded. After discarding them, the sample size was reduced to 94. Also, we note that since the measured values of LDH were very large, its logarithm was employed in the analysis. The descriptive statistics of 94 subjects for the different variables, such as percentages, means, and standard deviations (SD) to describe the sample were utilized.

In the first step of data modelling, we performed latent class model on latent disease status and LDH, UA, and Cr measures to correctly classify subjects into meaningful subgroups. The Bayesian approach was applied to estimate the model parameters and subsequently Sensitivity, Specificity, and AUC. To obtain the Bayesian estimated parameters, the Markov chain Monte Carlo (MCMC) techniques according to the posterior distribution was employed. Likewise, uninformative priors for all the parameters were utilized. Posterior inference was done by calculating mean, median, SD, and 95% credible interval (CrI) of all the parameters. For analysis, first, the presented model for each the chemical continuous factor by Choi *et al*.^39^ was considered.

Latent class model for each serum marker. Assume that there are *n* participants in the sample. Let *Y*_*i*_ denotes the diagnostic results of the three tests (LDH, UA, and Cr) for *i*^th^ patient. Let the binary variable *D*_*i*_ denote the true condition for *i*^th^ transplant patient, which is not observed (*D*_*i*_ = 1 for disease present and *D*_*i*_ = 0 for disease absent). Let *π*_*i*_ denote the probability that the *i*^th^ patient is diseased. The model can be written in the following hierarchical form:

1 $$\begin{array}{c}{D}_{i}\sim Bernoulli({\pi }_{i})\\ ({Y}_{i}|{D}_{i})\sim {p}_{1}{(\cdot |{\mu }_{{D}^{+}},{\sigma }_{{D}^{+}}^{2})}^{{D}_{i}}{p}_{2}{(\cdot |{\mu }_{{D}^{-}},{\sigma }_{{D}^{-}}^{2})}^{1-{D}_{i}}\end{array}$$

where *μ*_D+_ and ${\mu }_{{D}^{-}}$ are the means, and ${\sigma }_{{D}^{+}}^{2}$ and ${\sigma }_{{D}^{-}}^{2}$ are the variances for diseased (*D*^+^) and non-diseased (*D*^−^) populations, respectively. Also, *p*_1_(·) is the $N({\mu }_{{D}^{+}},{\sigma }_{{D}^{+}}^{2})$ probability density function (pdf) and *p*_2_(·) is the $N({\mu }_{{D}^{-}},{\sigma }_{{D}^{-}}^{2})$ pdf. After obtaining the estimation of ${\mu }_{{D}^{+}}$, ${\sigma }_{{D}^{+}}^{2},{\mu }_{{D}^{-}}$, and ${\sigma }_{{D}^{-}}^{2}$, the sensitivity, specificity, and AUC were calculated. The sensitivity and specificity of each diagnostic test in detecting GVHD for cut-off points values ${c}_{1}\,{\rm{and}}\,{c}_{2}\in (\,-\,\infty ,\infty )$, defined as: sensitivity = $P(Y > {c}_{1})=1-\Phi (\frac{{c}_{1}-{\mu }_{{D}^{+}}}{\sqrt{{\sigma }_{{D}^{+}}^{2}}})$, specificity = $P(Y\le {c}_{2})=\Phi (\frac{{c}_{2}-{\mu }_{{D}^{-}}}{\sqrt{{\sigma }_{{D}^{-}}^{2}}})$. It is of interest to note that, the cut-points *c*_1_ and *c*_2_ were calculated according to the formula presented by Choi *et al*. For choosing the optimal cut-off point c, the Youden index, which is the most popular one in practice, was utilized. This index is formulated as J = sensitivity + specificity − 1 that is equivalent to the maximum sum of sensitivity and specificity across all possible cut-off point values. Based on optimal cut-off point, the Sensitivity and Specificity were determined. Lastly, the AUC which is the probability that the value of the diagnostic test in a randomly chosen positive subject will be higher than the value of the marker in a randomly chosen negative subject can be calculated as: ${\rm{AUC}}=\Phi (-\frac{{\mu }_{{D}^{-}}-{\mu }_{{D}^{+}}}{\sqrt{{\sigma }_{{D}^{+}}^{2}+{\sigma }_{{D}^{-}}^{2}}})$ such that Φ is the cumulative distribution function of a standard normal for the test’s results. The receiver operating characteristic (ROC) curve based on single diagnostic test can be constructed by plotting (1-specificity, sensitivity). Notably, to determining how close the distribution of *Y*_*D*_^+^ to the distribution of *Y*_*D*_^−^, the measure Δ was utilized. Clearly, if the overlap between the distribution of diseased and non-diseased groups become large, assigning the true disease status in the overlapping region will be difficult^39^. For each of the three models, one chain was run for 20,000 iterations with lag 25. The first 8,000 iterations were discarded. Convergence of the MCMC chains was evaluated using Geweke statistic and autocorrelation plots.

Latent class analysis for combining serum markers. Various combinations (pairwise and three) of multiple biomarker results into a single test were considered. First, the discriminant ability of the combinations without covariate adjustment was examined. Second, we evaluated the diagnostic accuracy of the combinations with controlling for some covariates. Initially, binary logistic and regression models was applied to explore significant covariates. Evidently, covariates which are associated with GVHD and considered biomarkers at significance level of 20% were incorporated in latent class models. Indeed, the significance level of 20%, rather than 5% was utilized in selecting covariates. Results (not shown) exhibited that age, DP-gender, GVHD prophylaxis, and conditioning regimen are significant covariates in either logistic or regression models. Consequently, the latent class model according to proposed model by Yu *et al*.^40^ is specified as:

2 $$\begin{array}{l}{D}_{i}|{{\rm{x}}}_{i} \sim Bernoulli({\pi }_{i})\\ {Y}_{i}|{{\rm{x}}}_{i},{D}_{i} \sim {\rm{M}}{\rm{V}}{\rm{N}}(\mu ({{\rm{x}}}_{i},{D}_{i}),{\Sigma }_{{D}_{i}})\end{array}$$

where *Y*_*i*_ = (*Y*_*i*1_, *Y*_*i*2_, …, *Y*_*ik*_)′ denote the vector of *k* correlated diagnostic tests. The covariate vector is denoted by x. ∑_*D*_ denote the covariance matrix of the test results. The $\mu ({x}_{i},{D}_{i})$ for *k*th test value is defined as: ${\mu }_{k}({{\rm{x}}}_{i},{D}_{i})={\beta }_{0}^{(k)}+{\beta }_{x}^{(k)}{{\rm{x}}}_{i}+{\beta }_{D}^{(k)}{D}_{i}+{\beta }_{xD}^{(k)}({{\rm{x}}}_{i}{D}_{i})$ where ${\beta }^{(k)}=({\beta }_{0}^{(k)},{\beta }_{x}^{(k)},{\beta }_{D}^{(k)},{\beta }_{xD}^{(k)})$ is the row vector of regression coefficients. Of note, to model the covariate effects on the disease status, a logistic model was used as: $\log \,\frac{P({D}_{i}=1)}{1-P({D}_{i}=1)}={\alpha }_{0}+{\alpha }_{1}{{\rm{x}}}_{i1}+\cdots +{\alpha }_{j}{{\rm{x}}}_{ij}$, such that α_0_ is the baseline and α_1_, …, α_j_ are unknown regression coefficients. After estimation of the parameters, the diagnostic accuracy measures based on linear combination of the tests (Ycombined = αY) for a given cut-off point value c for plotting combined ROC (cROC) can be estimated as: ${\rm{csensitivity}}({\rm{x}})=\Phi (\frac{a{\prime} \mu ({\rm{x}},1)-{c}_{1}^{\ast }}{\sqrt{a{\prime} {\Sigma }_{1}a}}),$ ${\rm{cspecificity}}({\rm{x}})=\Phi (\frac{{c}_{2}^{\ast }-a{\prime} \mu ({\rm{x}},0)}{\sqrt{a{\prime} {\Sigma }_{0}a}}),$ ${\rm{cAUC}}({\rm{x}})=\Phi (\sqrt{a{\prime} \varDelta (x)}),$such that $\Delta ({\rm{x}})=\mu ({\rm{x}},1)-{\rm{\mu }}({\rm{x}},0)$ and $a={({\Sigma }_{0}+{\Sigma }_{1})}^{-1}\Delta ({\rm{x}})$. It is important to note that the calculation of cut-off points ${c}^{\ast }=(c1,{c}_{2}^{\ast })$ for a composite test is as the same as a diagnostic test. For choosing optimal cut-off point, the Youden index which mentioned before, was used for the combinations. Afterward, the cSensitivity and cSpecificity were selected. For the models, the convergence of the MCMC chains for all the LCMs (with and without covariate adjustment) was assessed using autocorrelation plots. Furthermore, the convergence was obtained by running one chain with 35,000 iterations with lag 45. The first 17,000 iterations were discarded.

The Bayesian latent class models were fitted by using OpenBUGS 3.2.1^41^. The R package R2OpenBUGS^42^ also applied as an interface between R 3.6.2^43^ and OpenBUGS. After determining the GVHD groups based on optimal composite test, in order to evaluate the association between each of continuous variables and GVHD, an independent sample t-test was performed and chi-square test was applied for each categorical variable. All comparisons were two-tailed and p-value of less than 0.05 was taken as statistically significant. Descriptive analyses were performed using R version 3.6.2.

## Data Availability

The datasets analysed during the current study are not publicly available due to the reasonable risk that study participants may be identified. The datasets presented in this study may be available from the corresponding author on reasonable request.
